# Relationships and mediating mechanisms between university teachers’ marital relationship, work values, and social support on job satisfaction

**DOI:** 10.3389/fpsyt.2025.1665141

**Published:** 2025-09-09

**Authors:** Linlin Feng, Xiaohan Zhang, Hao Zhong, Wenqing Zhang

**Affiliations:** School of Marxism, Shandong University of Technology, Zibo, China

**Keywords:** university teachers’ job satisfaction, marital relationship, work values, social support, mediating mechanisms, strategies for improvement

## Abstract

Job satisfaction is a critical factor that can be used to estimate the quality of university teachers’ work, and it affects teachers’ career development as well as the students’ mental health. In this study, we surveyed 456 university teachers by using demographic characteristics information questionnaire, marital relationship questionnaire, positive and negative affect schedule, work values inventory, work performance questionnaire, social support rating scale, and teacher job satisfaction questionnaire. We investigated the factors including marital relationship, work values, and social support affecting university teachers’ job satisfaction and explored strategies for its improvement. The results revealed that university teachers’ job satisfaction was relatively high, and male teachers received significantly higher scores in the working environment as well as development and promotion dimensions than females. Marital relationship was directly and positively associated with job satisfaction and indirectly correlated with it through positive affect experienced in the marriage; work values were directly and positively associated with job satisfaction and indirectly correlated with it through work performance; objective support and utilization of support were directly and positively associated with job satisfaction and indirectly correlated with it through subjective support. Therefore, satisfactory marital relationship, positive work values orientation, more objective social support, and better utilization of support were crucial factors influencing university teachers’ job satisfaction. Moreover, positive affect experienced in the marriage, work performance, and subjective support were also critical. This study identifies areas for change which may improve university teachers’ job satisfaction.

## Introduction

Job satisfaction is an essential parameter for measuring university teachers’ work quality (1). Those with higher levels of job satisfaction typically demonstrate more enthusiasm and are more active in their work (2), whereas those with lower levels of job satisfaction tend to experience burnout and make career changes (3). Teachers’ job satisfaction is closely related to teachers’ professional development, students’ development, and the improvement of education as well as teaching quality (4). The significance of understanding university teachers’ job satisfaction has grown in recent years, especially as it directly impacts the quality of higher education and students’ learning experiences. However, the review of the literature reveals that existing studies have mainly concentrated on internal job-related factors, for example, work pressure and average daily workload negatively affect teachers’ job satisfaction (5). Moreover, the ratio of teaching time investment and scientific research time investment both affect job satisfaction (6). This oversight creates a significant gap in our understanding, as family is an important part of individual’s life and may have a profound impact on job satisfaction. Therefore, the factors influencing university teachers’ job satisfaction and strategies for improvement warrant investigation.

The field of individual’s relationship mainly includes personal field and social field. For employees, the most important personal field is family, and the most important social field is work. Support from both the family field and the work field will have positive impacts on employees’ work attitude and behavior (7, 8), so that employees can be better engaged in their work (9). On the family field, the marital relationship is the foundation and source of the familial relationship, good marital relationship has positive influences on individual’s job satisfaction (10). On the work field, there is a significant relationship between job values and job satisfaction, and job values can significantly predict job satisfaction (11). In addition, personal development depends on social relationships. The help and support individuals receive from social relationships and interactions are referred to as social support (12). Social support plays a crucial role in the development of individual’s health and ability to respond to various adverse stimuli. There is a significant relationship between subjective support and objective support as well as the degree of burnout (13). Accordingly, the factors affecting university teachers’ job satisfaction from the marital relationship, work values, and social support were examined.

## Marital relationship and university teachers’ job satisfaction

The relationships among family members, particularly the marital relationship, affect both the emotional well-being in the family and the interpersonal activity as well as activity efficiency in the workplace (14). For example, Karatepe and Kilic (15) found that spouse support was positively correlated with employees’ physical condition and job satisfaction, that is, the better the husband’s and wife’s relationship was, the better the employees’ physical condition and job satisfaction would be. Therefore, a satisfactory marital relationship can increase university teachers’ job satisfaction. Different types of marital relationships cause different emotional responses. An intimate and harmonious husband-wife relationship generates positive affect in the form of affection and fondness, but a relationship of estrangement produces negative affect in the form of contempt and hatred (16).

Research has indicated that emotional responses significantly affect employees’ job satisfaction (17). Positive affect can help employees effectively deal with problems and difficulties at work, thereby promoting career development. Negative affect, however, has the opposite effect on job satisfaction. Negative affect causes employees to develop negative tendencies at work, reduces their enthusiasm towards work, and decreases job satisfaction (18). It was found that work-family conflicts had significant impacts on satisfaction (19). However, there are few studies on the impact of the marital relationship on job satisfaction. If the impact of the marital relationship on job satisfaction can be confirmed, it can provide more demographic support for the marital relationship to affect individuals’ job satisfaction. Therefore, we investigated the effect of marital relationships on university teachers’ job satisfaction and the mediating effects of both positive and negative emotional responses.

## Work values and university teachers’ job satisfaction

Work values are individuals’ beliefs and attitudes towards their job or the value orientation they exhibit throughout their career (20). Work values are regarded as indicators to predict work behaviors and results including work performance (21). It can be used to measure employees’ judgements of their work, which determine their career choices and development, as well as affect their work attitude, behavior, and performance (22). Previous studies have demonstrated that job satisfaction at work is positively predicted by work values (23), and the higher their work values are, the higher their work performance is (24). Job satisfaction relates to employees’ attitudes and their judgement of their job and judgement of working environment (25). Work values, a type of stable value judgement and evaluation system, affect job satisfaction, and they were closely related (26, 27).

Work performance elicits positive evaluations from others, enables employees to establish friendly relationships with their colleagues, and increases job satisfaction (28). Based on motivation and expectations, employees’ job satisfaction is based on expectations and the rewards generated from their performance (29), that is, internal and external rewards (e.g., salary, organizational recognition, and positive experience). Liu et al. (30) found that job satisfaction was positively correlated with job performance (30). These rewards tied to employees’ performance increase their job satisfaction (31). Therefore, we studied the effect of work values on job satisfaction and the mediating effect of work performance.

## Social support and university teachers’ job satisfaction

Social support can alleviate individuals’ stress at work and the negative affect caused by stress, thereby allowing employees to form positive work attitudes and behavioral tendencies (32). From the function of social support, social support can be divided into objective and subjective support. Objective support refers to the direct assistance through materials, participation in social networks, and group relationships. Objective support is independent from individuals’ subjective feelings. Subjective support refers to the emotional support individuals receive, including feelings of being respected, supported, and understood in society. Xiao (33) proposed another dimension of social support, the utilization of support. The utilization of support refers to the individual’s use of social support. Studies have confirmed the effect of social support on job satisfaction. For example, Zhu and Cheng (34) found that social support can directly and positively predict teachers’ professional identity and job satisfaction, and social support was significantly and positively associated with job satisfaction. However, few studies have emphasized the internal relationships within social support and the mechanism by which these relationships affect job satisfaction.

Deng et al. (35) discovered that objective support, subjective support, and utilization of support directly affect employees’ job satisfaction. Although objective and subjective support differ, they are closely correlated. Research has indicated a significant correlation between objective and subjective support (36). Individuals should be able to experience objective support emotionally so as to experience subjective support. Therefore, the present study assumed that objective support directly affects job satisfaction, and indirectly affects job satisfaction through subjective support. Some individuals, while being able to receive support, tend to reject assistance; consequently, they cannot effectively utilize social support to help themselves (33). Hence, the utilization of support is a crucial factor affecting the individual’s experience of emotional support and job satisfaction.

Therefore, marital relationship, work values, and social support are key factors that affect university teachers’ job satisfaction. We examined the effects of the marital relationship, work values, and social support on university teachers’ job satisfaction and the mechanisms of these three influential factors. On the basis of the studies described above, we made the following hypotheses: (1) marital relationships positively predict job satisfaction, and the emotional responses in marriage have mediating effects; (2) work values positively predict job satisfaction, and work performance has a mediating effect; and (3) objective support and utilization of support positively predict job satisfaction, and subjective support has mediating effects. Although previous studies have discussed the marital relationship (37), work values (38) and social support (39) on individual’s job satisfaction to varying degrees, few studies have discussed the three variables together. Individual’s development is inseparable from family, work and society. The marital relationship corresponds to family, work values correspond to work, and social support corresponds to social relations. Therefore, this study combines the marital relationship, work values and social support, which can provide empirical support in different aspects for improving university teachers’ job satisfaction.

## Methods

### Participants and procedure

In this cross-sectional study by means of an online questionnaire survey, convenience cluster sampling was used. A power analysis was conducted using G^*^Power 3.1 (40) in which the estimated effect size of r = 0.21, α = 0.05 (two-tailed), and power = 0.80. The result suggested a required sample size of *N* = 136. We recruited 500 university teachers from universities in Shandong province and distributed 500 questionnaires. After invalid questionnaires were excluded because there were much missing data, a total of 456 valid questionnaires were returned, with a recovery rate of 91.20%. Among the participants, 248 were men, and 208 were women. The age range of the participants was between 24 and 55 years, with an average age of 37.40 ± 5.25 years.

The study was carried out as follows. First, we communicated with the university teachers on the Internet. After obtaining their consent, we sent questionnaires online. After obtaining their informed consent, they answered the online questionnaires. All these participants were willing to cooperate with this survey and can complete our questionnaires independently. The measures were administrated to the participants by trained research assistants online. The data collection procedures lasted approximately 40 minutes.

The participants completed measures including the demographic characteristics information questionnaire, marital relationship questionnaire, positive and negative affect schedule, work values inventory, work performance questionnaire, and teacher job satisfaction questionnaire (total 120 items). The participants were informed that the measures included questions on their beliefs and experiences in daily life and were encouraged to respond to all the items accurately. Upon study completion, each participant received a bonus (CNY 12 = USD 1.667) for compensation.

### Ethical statements

This study was conducted under the approval of the Institutional Review Board (IRB) at Shandong University of Technology. The participants were made aware of the voluntary and confidential nature of this study. They were fully informed of the research before participation, such as purpose and content. Written consent was obtained prior to the administration, all participants were over 18 years old. This study caused no harm to participants’ physical and mental health, and the results of this study were maintained confidentially.

## Measures

### Marital relationship

University teachers’ marital relationship was investigated by the marital relationship questionnaire developed by Chen et al. (41). The questionnaire consisted of 14 items and was divided into three dimensions: couple communication (four items, e.g., ‘I do not think it is necessary to tell my spouse about my feelings directly because he or she should be able to feel them’), couple affect (five items, e.g., ‘I believe my spouse will treat me well and will not do anything to harm me’), and couple cognition (five items, e.g., ‘I know the discomfort and illnesses my spouse has’). The items were scored from 1 (*strongly disagree*) to 5 (*strongly agree*). Response scores were averaged to create their composite scores for data analysis. Those who received a higher score had a more satisfactory marital relationship. The α coefficient of the overall questionnaire was 0.84, and that of the three dimensions was 0.72, 0.86, and 0.71, respectively. The confirmatory factor analysis of the questionnaire showed that the goodness-of-fit was satisfactory: *χ^2^/df*=2.951, RMSEA=0.065, NFI=0.93, IFI=0.95, TLI=0.93, CFI=0.95.

### Emotional responses

University teachers’ emotional responses in their marital relationship were estimated by the positive and negative affect schedule developed by Huang et al. (42). The schedule consisted of 20 items classified into two dimensions: positive affect (ten items, e.g., ‘I feel interested’) and negative affect (ten items, e.g., ‘I feel annoyed’). The items were scored from 1 (*never*) to 5 (*always*). Response scores were averaged to create their composite scores for data analysis. Those who received a higher score in the positive affect dimension had stronger positive affect, whereas those who received a higher score in the negative affect dimension had stronger negative affect. The α coefficient of the overall schedule was 0.69, and that of the two dimensions was 0.87 and 0.83, respectively. The confirmatory factor analysis of the schedule showed that the goodness-of-fit was satisfactory: *χ^2^/df*=2.685, RMSEA=0.061, NFI=0.95, IFI=0.97, TLI=0.95, CFI=0.97.

### Work values

University teachers’ work values were explored by the work values inventory developed by Xu (43). The inventory consisted of 27 items and was divided into seven dimensions: material rewards (four items, e.g., ‘The job guarantees a high pay and many benefits’), reputation and social status (four items, e.g., ‘Teachers have a high social status’), career development (four items, e.g., ‘The school enables me to conduct scientific research’), interpersonal relationship (four items, e.g., ‘Coworkers share the results of their work with each other’), organizational management (four items, e.g., ‘The school creates a comfortable working environment’), altruistic dedication (four items, e.g., ‘Teachers care for the students’), safety and stability (three items, e.g., ‘Being a teacher is a relatively stable job’). The items were scored from 1 (*highly unimportant*) to 5 (*highly important*). Response scores were averaged to create their composite scores for data analysis. Those who received a higher score had higher work values. The α coefficient of the overall inventory was 0.89, and that of the seven dimensions was 0.61, 0.77, 0.66, 0.77, 0.65, 0.68, and 0.68, respectively. The confirmatory factor analysis of the inventory showed that the goodness-of-fit was satisfactory: *χ^2^/df*=2.347, RMSEA=0.054, NFI=0.91, IFI=0.95, TLI=0.90, CFI=0.94.

### Work performance

University teachers’ work performance was evaluated by the work performance questionnaire developed by Ma (44). The questionnaire consisted of 14 items and was categorized into three dimensions: task performance (four items, e.g., ‘I comply with school rules and regulations’), interpersonal promotion (five items, e.g., ‘I have a healthy relationship with my coworkers’), and work devotion (five items, e.g., ‘I am willing to undertake challenging tasks’). The items were scored from 1 (*strongly disagree*) to 6 (*strongly agree*). Response scores were averaged to create their composite scores for data analysis. Those who received a higher score had superior work performance. The α coefficient of the overall questionnaire was 0.90, and that of the three dimensions was 0.76, 0.82, and 0.86, respectively. The confirmatory factor analysis of the questionnaire showed that the goodness-of-fit was satisfactory: *χ^2^/df*=2.448, RMSEA=0.056, NFI=0.95, IFI=0.97, TLI=0.96, CFI=0.97.

### Social support

University teachers’ received and perceived social support was assessed by the social support rating scale developed by Xiao (33). The scale comprised 10 items and was classified into three dimensions: subjective support (four items, e.g., ‘How many close friends do you have, from whom you can receive support and assistance?’), objective support (three items, e.g., ‘What were the sources of financial support and assistance you have used to solve problems?’), and utilization of support (three items, e.g., ‘What do you do when you have worries?’). The items were scored based on respondents’ selections for each item. The α coefficient of the overall scale was 0.84, and that of the three dimensions was 0.76, 0.78, and 0.74, respectively. The confirmatory factor analysis of the scale showed that the goodness-of-fit was satisfactory: *χ^2^/df*=2.540, RMSEA=0.058, NFI=0.95, IFI=0.97, TLI=0.94, CFI=0.97.

### Job satisfaction

University teachers’ job satisfaction was measured by the job satisfaction questionnaire developed by Shu (45). The questionnaire comprised 30 items categorized into five dimensions: teaching (six items, e.g., ‘I feel a sense of accomplishment at work’), working environment (six items, e.g., ‘I am satisfied with my interpersonal relationship with my coworkers’), development and promotion (six items, e.g., ‘I think there are plenty of opportunities for teachers to further their studies’), welfare and remuneration (six items, e.g., ‘I think the job as a teacher guarantees a secure livelihood’), and university president management (six items, e.g., ‘I think the president understands that teaching is hard work’). The items were scored from 1 (*strongly disagree*) to 5 (*strongly agree*). Response scores were averaged to create their composite scores for data analysis. Those who received a higher score had higher job satisfaction. The α coefficient of the overall questionnaire was 0.94, and that of the five dimensions was 0.77, 0.73, 0.72, 0.84, and 0.89, respectively. The confirmatory factor analysis of the questionnaire showed that the goodness-of-fit was satisfactory: *χ^2^/df*=2.907, RMSEA=0.065, NFI=0.91, IFI=0.94, TLI=0.90, CFI=0.93.

### Statistical analysis

The data were proposed and analyzed using SPSS (version 26.0) and Amos (version 23.0). Amos 23.0 was used to conduct path analysis with maximum likelihood estimation. Evaluations of Structural Equation Modeling were conventionally based on the following statistics: the normed fit index (NFI), the incremental fit index (IFI), the Tacker-Lewis index (TLI), the comparative fit index (CFI), and the root mean square error of approximation (RMSEA) (46, 47). Moreover, the Bootstrap method was used to test the mediating effect, wherein repeated random sampling was used to collect *n* samples (*n* = 5000) from the raw data to generate and save *n* mediating effect values to form an approximate sampling distribution, and the mean path coefficient of the mediating effect was calculated. If the 95% confidence interval of these mean path coefficients did not include 0, it indicated that the mediating effect was significant (48).

Harman single factor test was used to analyze all the items in the questionnaire together to test the common method deviation (49). The results showed that there were 28 factors with eigenvalue greater than 1 without rotation, and the first factor variation explanation rate was 23.62%, which was less than the critical standard of 40%, indicating that the common method deviation problem was not obvious.

## Results

### Gender differences of university teachers’ job satisfaction

To understand the overall situation of university teachers’ job satisfaction, we performed a descriptive statistical analysis on their satisfaction, and presented the means and standard deviations of its dimensions. The means of job satisfaction and the means of its dimensions were significantly higher than 3 (the intermediate critical value) (*ps* < 0.001), indicating that university teachers had a relatively high level of job satisfaction. To determine whether job satisfaction differed between genders, we used gender as the independent variable and the scores for job satisfaction and its dimensions as dependent variables for an independent samples *T* test. The results (**Table 1**) indicated differences between genders only in the working environment as well as in the development and promotion dimensions (*t* = 2.03, *p* < 0.05; *t* = 2.18, *p* < 0.05). The male teachers received significantly higher scores in the two dimensions than did the female teachers.

**Table 1:** Descriptive statistics and gender differences of job satisfaction.

Variable	Male teachers		Female teachers		*t*
	*M*	*SD*	*M*	*SD*	
Job satisfaction	3.98	0.44	3.90	0.48	1.30
Teaching	4.12	0.49	4.05	0.49	1.09
Working environment	3.92	0.46	3.79	0.49	2.03^*^
Development and promotion	4.01	0.45	3.87	0.52	2.18^*^
Welfare and remuneration	4.05	0.54	3.99	0.55	0.82
University president management	3.78	0.60	3.78	0.64	-0.05

^*^ *p* < 0.05.

### Correlation analysis

We performed a Pearson correlation analysis on each variable. The results (**Table 2**) indicated that marital relationship, positive affect, work values, work performance, objective support, utilization of support, and subjective support had significantly positive correlations with job satisfaction (*p*s < 0.01); negative affect had a significantly negative correlation with job satisfaction (*p* < 0.001). Marital relationship was significantly and positively correlated with positive affect (*p* < 0.001), but significantly and negatively correlated with negative affect (*p* < 0.001); work values were significantly and positively correlated with work performance (*p* < 0.001); objective support and utilization of support were significantly and positively correlated with subjective support (*p*s < 0.001).

**Table 2:** Correlations among the key study variables.

Variable	1	2	3	4	5	6	7	8	9
1 Marital relationship	–								
2 Positive affect	0.49^***^	–							
3 Negative affect	-0.54^***^	-0.34^***^	–						
4 Work values	0.47^***^	0.43^***^	-0.25^***^	–					
5 Work performance	0.47^***^	0.44^***^	-0.29^***^	0.66^***^	–				
6 Objective support	0.19^**^	0.30^***^	-0.11	0.28^***^	0.36^***^	–			
7 Utilization of support	0.19^**^	0.27^***^	-0.12	0.26^***^	0.36^***^	0.37^***^	–		
8 Subjective support	0.42^***^	0.49^***^	-0.26^***^	0.48^***^	0.50^***^	0.59^***^	0.48^***^	–	
9 Job satisfaction	0.56^***^	0.57^***^	-0.33^***^	0.57^***^	0.48^***^	0.31^***^	0.35^***^	0.47^***^	–
*M*	4.13	3.64	1.84	4.24	4.94	1.64	2.81	6.31	3.94
*SD*	0.48	0.53	0.45	0.36	0.56	0.27	0.62	0.99	0.46

^**^ *p* < 0.01, ^***^ *p* < 0.001.

### Relationships between marital relationship, work values, social support and university teachers’ job satisfaction

We examined the relationships between family, work values, and social support on university teachers’ job satisfaction. In terms of family, we examined the correlation of marital relationship on job satisfaction and the mediating effects of emotional responses. In terms of work values, we examined the correlation of work values on job satisfaction and the mediating effect of work performance. In terms of social support, we examined the correlation of objective support and the utilization of support on job satisfaction and the mediating effects of subjective support. The results (**Table 3**) revealed that in terms of family, marital relationship was significantly and positively associated with positive affect and job satisfaction, and was significantly and negatively associated with negative affect; positive affect was significantly and positively associated with job satisfaction. However, the relationship of negative affect on job satisfaction was not significant. Positive affect had a partial mediating effect between marital relationship and job satisfaction. On the other hand, negative affect did not have a significant mediating effect. In terms of work values, work values were significantly and positively associated with work performance and job satisfaction; work performance was significantly and positively associated with job satisfaction. A partial mediating effect of work performance between work values and job satisfaction was identified. In terms of social support, objective support and utilization of support were significantly and positively associated with subjective support; subjective support was significantly and positively associated with job satisfaction. The correlation of objective support to job satisfaction was not significant. However, the correlation of utilization of support to job satisfaction was significant and positive. Subjective support had a complete mediating effect between objective support and job satisfaction, and had a partial mediating effect between utilization of support and job satisfaction.

**Table 3:** Regression analysis of influencing factors of job satisfaction.

Regression equation		Fitting index			Coefficient significance	
Dependent variable	Independent variable	*R*	*R^2^*	*F*	*β*	*t*
Job satisfaction		0.56	0.32	104.50***		
	Marital relationship				0.56	10.22***
Positive affect		0.49	0.24	72.32***		
	Marital relationship				0.49	8.50***
Negative affect		0.54	0.29	90.97***		
	Marital relationship				-0.54	-9.54***
Job satisfaction		0.65	0.43	55.82***		
	Marital relationship				0.37	5.71***
	Positive affect				0.38	6.57***
	Negative affect				-0.004	-0.07
Job satisfaction		0.57	0.32	108.14***		
	Work values				0.57	10.40***
Work performance		0.66	0.43	170.15***		
	Work values				0.66	13.04***
Job satisfaction		0.59	0.34	58.56***		
	Work values				0.45	6.29***
	Work performance				0.18	2.53*
Job satisfaction		0.40	0.16	21.91***		
	Objective support				0.21	3.24**
	Utilization of support				0.27	4.18***
Subjective support		0.65	0.42	82.92***		
	Objective support				0.47	8.72***
	Utilization of support				0.31	5.62***
Job satisfaction		0.49	0.24	23.87***		
	Objective support				0.04	0.51
	Utilization of support				0.16	2.40*
	Subjective support				0.37	4.84***

^*^ *p* < 0.05, ^**^ *p* < 0.01, ^***^ *p* < 0.001.

All analysis controlled for gender and age to account for demographic influences.

**Figure 1:**
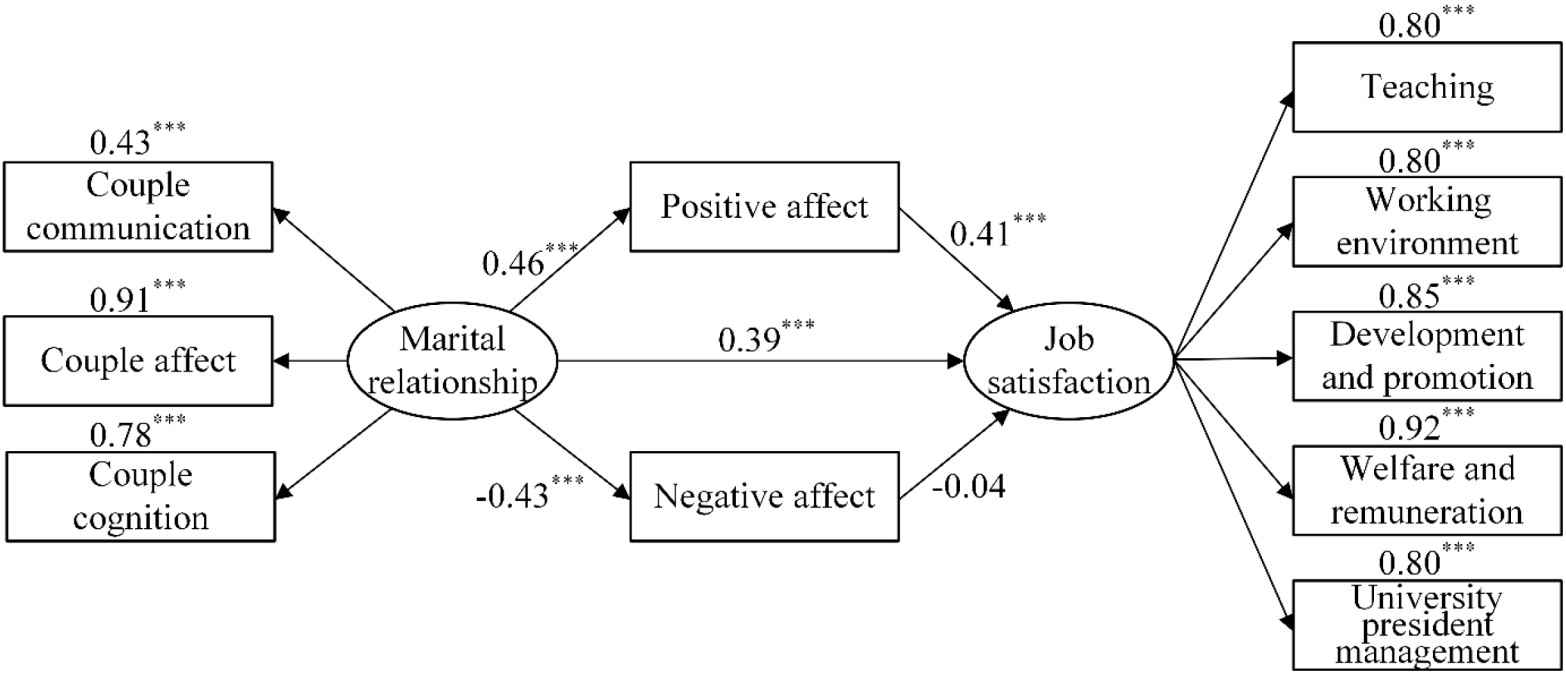
The relationships between marital relationship, positive affect, negative affect, and job satisfaction. ^***^ *p* < 0.001.

**Figure 2:**
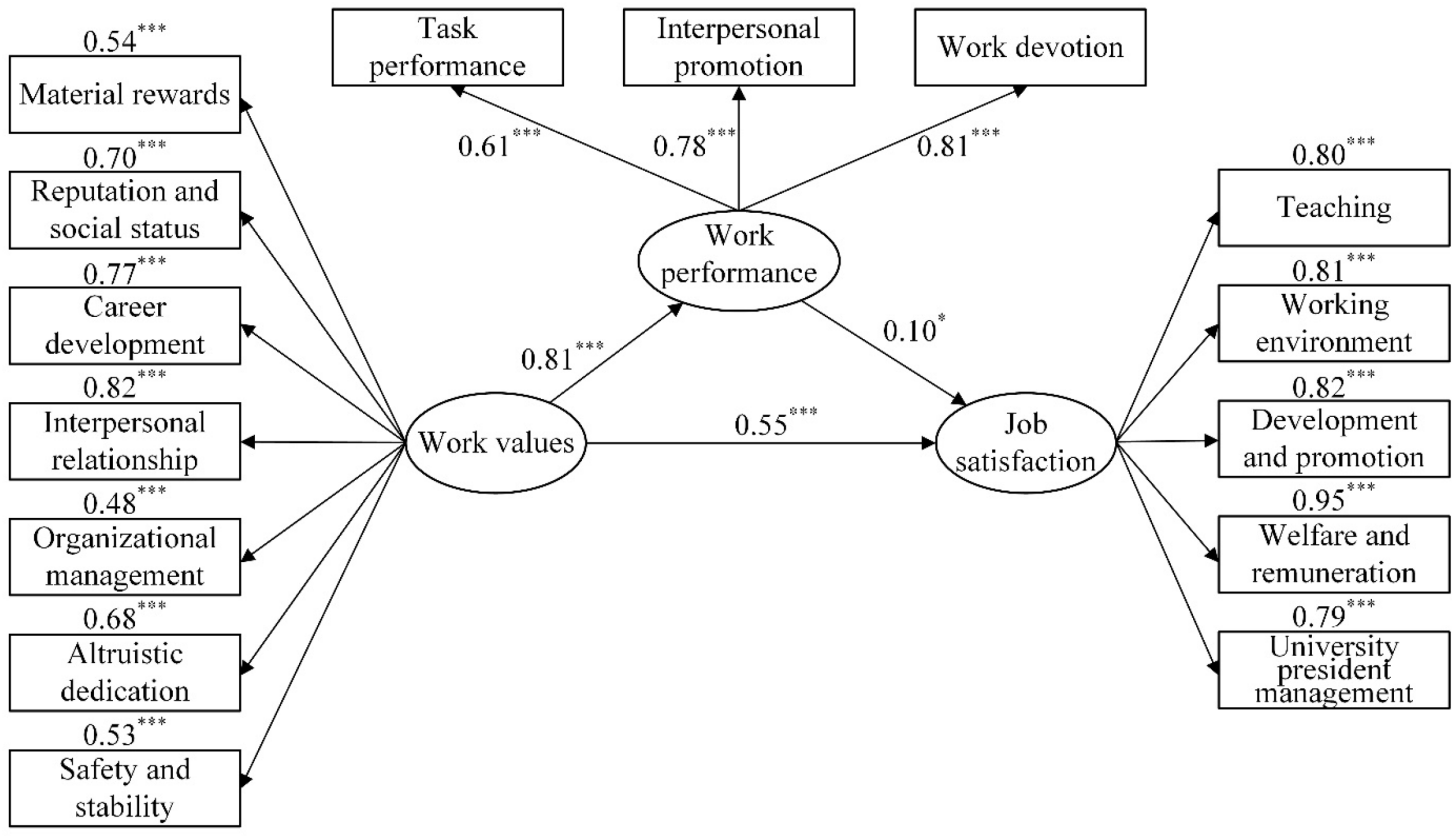
The relationships between work values, work performance, and job satisfaction. ^*^ *p* < 0.05, ^***^ *p* < 0.001.

**Figure 3:**
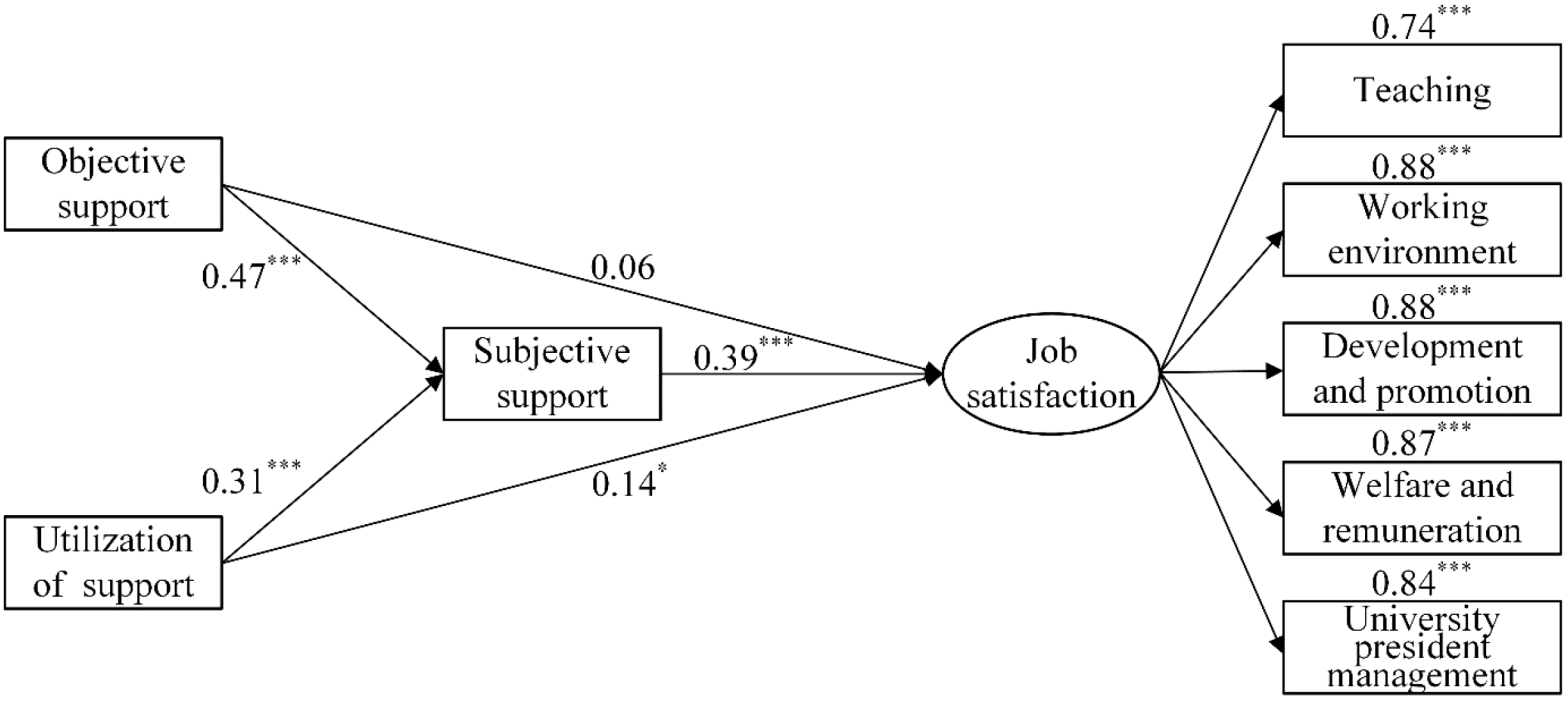
The relationships between objective support, utilization of support, subjective support, and job satisfaction. ^*^ *p* < 0.05, ^***^ *p* < 0.001.

Structural equation models M_1_ (**Figure 1**), M_2_ (**Figure 2**), and M_3_ (**Figure 3**) were constructed to analyze the mediating effects of emotional responses between marital relationships and job satisfaction, work performance between work values and job satisfaction, and subjective support among objective support, utilization of support, and job satisfaction. First, the results of the direct effect models indicated that the goodness-of-fit was satisfactory (*χ* ^2^/*df* < 2.32, RMSEA < 0.076, NFI > 0.94, IFI > 0.97, TLI > 0.95, CFI > 0.96). The direct effect of marital relationship on job satisfaction was significant (*β* = 0.57, *p* < 0.001); the direct effect of work values on job satisfaction was significant (*β* = 0.70, *p* < 0.001), and the direct effects of objective support and utilization of support on job satisfaction were significant (*β* = 0.35, *p* < 0.001; *β* = 0.33, *p* < 0.001). Second, the goodness-of-fit of the mediating effect models were also satisfactory (**Table 4**). According to the results, marital relationship was directly associated with job satisfaction and indirectly associated with it through positive affect, with the ratio of the mediating effect to the total effect being 32.60%, and the model explanatory power being 48.40%. Work values were directly associated with job satisfaction and indirectly associated with it through work performance, with the ratio of the mediating effect to the total effect being 12.84%, and the model explanatory power being 40.00%. Utilization of support was directly associated with job satisfaction, as well as objective support and utilization of support were associated with job satisfaction through subjective support, with the ratio of the mediating effect to the total effect being 46.34%, and the model explanatory power being 25.50%.

**Table 4:** Goodness-of-fit indices for the mediation models.

Model	*χ* ^2^	*df*	*χ* ^2^/*df*	RMSEA	NFI	IFI	TLI	CFI
M_1_	56.38	28	2.01	0.067	0.96	0.98	0.97	0.98
M_2_	176.09	74	2.38	0.078	0.92	0.95	0.93	0.95
M_3_	32.04	15	2.14	0.071	0.97	0.98	0.97	0.98

A percentile bootstrap method (the sampling frequency was 5,000 times, and a 95% confidence interval was used) and the PROCESS macro for SPSS developed by Hayes (50) was employed to analyze Model 4 (50). The mediating effects of emotional responses were examined using marital relationship as an independent variable, positive affect and negative affect as mediating variables, and job satisfaction as a dependent variable. The mediating effect of work performance was examined using work values as an independent variable, work performance as a mediating variable, and job satisfaction as a dependent variable. The mediating effect of subjective support was assessed using objective support and utilization of support as independent variables, subjective support as a mediating variable, and job satisfaction as a dependent variable. The results demonstrated that the 95% confidence interval used in the bootstrapping method to analyze the indirect effect of marital relationship on job satisfaction through positive affect did not contain 0, whereas that used to analyze the indirect effect through negative affect contained 0 (**Table 5**). The 95% confidence interval used to analyze the indirect effect of work values on job satisfaction through work performance did not contain 0 (**Table 5**). The 95% confidence interval used to analyze the indirect effect of objective support and utilization of support on job satisfaction through subjective support did not contain 0 (**Table 5**). Therefore, mediating effect of positive affect between marital relationship and job satisfaction, of work performance between work values and job satisfaction, and of subjective support among objective support, utilization of support, and job satisfaction were identified. All mediation models were analyzed using the PROCESS with gender and age included as covariates. This step aimed to rule out potential confounding effects of demographic variables on the relationships between the independent variables, mediators, and job satisfaction.

**Table 5:** Bootstrap analysis of the mediating effects.

	Effect value	Boot Standard error	Boot CI lower limit			Boot CI upper limit
Marital relationship → Job satisfaction	0.36	0.06	0.23			0.48
Marital relationship → Positive affect → Job satisfaction	0.18	0.05	0.10			0.29
Marital relationship → Negative affect → Job satisfaction	0.002	0.03	-0.06			0.07
Work values → Job satisfaction	0.58	0.09	0.40			0.76
Work values → Work performance → Job satisfaction	0.15	0.07	0.03			0.31
Objective support → Job satisfaction	0.01	0.01	-0.01			0.03
Utilization of support → Job satisfaction	0.04	0.02	0.01			0.07
Objective support → Subjective support → Job satisfaction	0.04	0.01	0.02			0.05
Utilization of support → Subjective support → Job satisfaction	0.05	0.01	0.03			0.07

All models controlled for gender and age to account for demographic influences.

## Discussion

### Gender differences in university teachers’ job satisfaction

The results suggested that university teachers had a relatively high level of job satisfaction. Additionally, except for the working environment as well as the development and promotion dimensions, no significant difference between the genders was discovered. The male teachers received higher scores in the two dimensions than did the female teachers; a possible explanation is that men and women have different social roles (51). Traditional society places higher expectations on men in terms of their career achievements. Traditional social culture endows men with higher career achievement expectations. Compared with men, women are in a disadvantaged position in many kinds of promotion opportunities in the workplace (52). As a result, women are less satisfied with the work environment and development promotion than men.

### Marital relationship and job satisfaction: the mediating effects of emotional responses

The study found that marital relationship was directly associated with job satisfaction. This is consistent with previous studies (53). The results showed that the higher the marital relationship quality of university teachers, the higher level of job satisfaction they experience, while the teachers who experienced more marital conflicts experienced lower job satisfaction. In addition, marital relationship exerts an indirect effect on job satisfaction via positive affect. Specifically, positive affect mediates this relationship, supporting our hypothesis. On the one hand, healthy marital relationship resulted in higher positive affect and job satisfaction. Couples with high marital satisfaction tended to express more positive affect, such as agreement, appreciation, and care for their relationship, whereas those with low marital satisfaction tended to exhibit more negative behavior, such as accusing or criticizing their spouses or suppressing their feelings (54).The positive emotional resources accumulated by the family (e.g., a relaxed and cheerful mood and a healthy family relationship) can improve employees’ performance at work (55). On the other hand, employees who experienced more positive affect in their families perceived that they were able to work happily and efficiently, exhibited more enthusiasm towards their work, and were more likely to experience a subjective sense of well-being and job satisfaction. Employees who experienced more positive family relationships derived more work-family gain and thus were more likely to express their happiness at work and maintain friendly relationships with their coworkers and supervisors. These employees were able to positively adjust their attitudes when encountering difficulties and setbacks at work, and overcome feelings of negative affect, thereby increasing their engagement at work and in their personal lives. This suggests that we need to increase positive affect and reduce the negative affect in the relationship of university teachers’ husband and wife through the corresponding coping strategies, so as to promote the participation of university teachers’ work role and improve their job satisfaction experiences.

This study did not find the mediating effects of negative affect between husband and wife relationship and job satisfaction. Tang (56) combed 151 literatures on teachers’ emotion management, and found that in the aspect of emotion regulation, most teachers will take the initiative to regulate their own emotions (56). Even teachers with negative emotions can make their own negative emotions not be brought into their work because of the nature of their education work. On the other hand, individuals with negative emotions usually strive to get rid of sad, angry, nervous and other bad feelings, so as to pursue happy and good positive experience. Therefore, individuals who experience negative emotions tend to make positive behaviors to alleviate bad emotions (57).

### Work values and job satisfaction: the mediating effect of work performance

This study found that work values were directly associated with job satisfaction, which is consistent with previous research (58). Work values are related to employees’ attitude, personal beliefs, and the goals they pursue at work. Work values are the fundamental factors determining job satisfaction (59–61). For example, when university teachers fulfil their work values, they develop a high level of enthusiasm towards their work and high level of job satisfaction. Conversely, when they fail to fulfil their work values, they have low level of job satisfaction (26).

We also found that work performance had a mediating effect between work values and job satisfaction, which was consistent with our research hypothesis. Work values influence employees’ cognition, affect, and behavior. When dealing with highly value-oriented matters, employees experience positive affect, which increases their resilience, encourages them to work hard, and improves their work performance. Therefore, the work values have a significant positive impact on their work input and work performance (62, 63). Employees with superior level of performance tend to positively evaluate their job and their coworkers’ achievements, thereby allowing them to maintain healthy relationships with their coworkers and feel more satisfied with their job (28, 64).

The fulfilment of work values were associated with work performance and job satisfaction, which is directly related to university teachers’ career development. Values are the basic factors that influence individuals to exert their subjective initiative; they affect how an individual functions in society (65). Therefore, the cultivation and improvement of work values are particularly crucial for university teachers. University teachers should properly orient their values, set clear goals at work, exhibit positive attitudes towards work, as well as pursue sense of accomplishment and job satisfaction through continuous hard work and progress. It is worth noting that the improvement of individual’s values depends not only on the expectations of the individuals but also on external factors, including the environment and organizational management. Kristof (66) proposed that the purpose of matching individual’s values is to emphasize the influence of the interaction between individuals and their environment on their attitude and behavior, thereby confirming the similarity between individual’s values and organizational values. In addition, the degree of matching of values influences employees’ mental status and behavior, value matching has a significant predictive effect on job satisfaction and turnover intention (67). A high degree of value matching between employees and the organizations they serve promote the exchange of information and improve behavioral coordination, thereby allowing employees to reach a consensus with their organizations in terms of beliefs and goals to benefit the organization (61). Accordingly, when promoting and establishing employees’ work values, organizations should conduct organizational culture training that informs employees of the organizational values, culture, and goals to increase the degree of value matching and create a win-win situation for both organizations and employees.

### Objective support, utilization of support and job satisfaction: the mediating effects of subjective support

The results of this study found that utilization of support was not only directly associated with job satisfaction, but also indirectly associated with job satisfaction through subjective support. Positive social support increases an individual’s happiness and satisfaction with their life. Conversely, a lack of social support creates a sense of alienation and isolation for individuals and reduces feelings of positive affect and happiness (68). With the continual development of education, university teachers have experienced stress resulting from scientific research, teaching, student-related factors, workload, and examinations. Teachers experience various types of stress. The subjective feelings teachers derive from social connections and their utilization of objective social support also vary. Some teachers receive support but reject help from others, whereas others may not know how to utilize external support and thus cannot effectively help themselves through social support, resulting in a decline in work performance and job satisfaction (69). Hence, to provide university teachers (especially those experiencing difficulties in life) with multilevel social support; specifically, to allow them to experience subjective support and to provide them with more mental and spiritual support so as to guide them to reasonably and adequately utilize the social support can substantially increase their sense of happiness.

We discovered that subjective support was significantly associated with job satisfaction, objective support was significantly associated with subjective support, whereas it was not associated with job satisfaction. Researchers generally agree emotional support is more impactful: perceived support, even if disconnected from objective reality, functions as psychological reality. It is this kind of psychological reality as an actual variable that affects people’s behaviors and development (70). University teachers’ income has increased, and their pursue for materiality is less strong as a result. Therefore, the effect of objective support on university teachers is indirect, which is one of the reasons why objective support was not associated with university teachers’ job satisfaction.

According to Maslow’s hierarchy of needs, an individual’s needs move from low-level physical needs to high-level mental and spiritual needs (71). In a society with a relative abundance in material conditions, university teachers are more eager to earn social respect and recognition than to receive material support, which increases their motivation towards work, encourages them to develop more positive attitudes, and facilitates the establishment of job identity, job satisfaction, and sense of self-realization. Accordingly, emotional experiences (e.g., feelings of being respected, supported, and understood) were more strongly correlated with their job satisfaction.

## Conclusion

A satisfactory marital relationship, a positive work value orientation, as well as more objective social support and better utilization of support were crucial factors affecting university teachers’ job satisfaction. To be specific: (1) Marital relationship was directly associated with job satisfaction. University teachers with satisfactory marital relationship had higher levels of job satisfaction, whereas those with unsatisfactory marital relationship had lower levels of job satisfaction. The positive affect of marriage had a mediating effect between martial relationship and job satisfaction; that is, marital relationship was indirectly associated with job satisfaction through the positive affect of marriage. (2) Work values were directly associated with job satisfaction. Specifically, the higher the work values of university teachers, the higher their job satisfaction. Work performance had a mediating effect between work values and job satisfaction, that is, job values were indirectly associated with job satisfaction through job performance. (3) Objective support and utilization of support were associated with subjective support. However, the objective support was not associated with job satisfaction. The higher the satisfaction level of the university teachers who can make full use of social support. Subjective support had mediating effects among objective support, utilization of support, and university teachers’ job satisfaction, that is, the utilization of support was indirectly associated with job satisfaction through subjective support.

## Implication, recommendation, and limitations

First, according to the martial relationship found in this study, it was significantly associated with the job satisfaction of university teachers. University teachers should maintain good and harmonious relationships between husband and wife, and promote the positive affect between husband and wife to flow into work, so as to feed the progress made in work back to the family and realize a virtuous circle. Schools should not only pay attention to teachers’ working status and performance in universities, but also pay attention to their family life, especially for teachers with difficulties in family life or problems in husband wife relationship, give care as much as possible, and provide spiritual and material support in time, so that they can be confident to face problems in family and work.

Second, according to the work values found in this study were significantly associated with the job satisfaction of university teachers. The competent education department should pay attention to the impact of teachers’ work values on their career development, and realize that the key to the improvement of education quality lies in the formation and cultivation of teachers’ work values and the improvement of work performance. Therefore, we should fully create conditions to provide teachers with a stage for promotion and talent display, so that teachers can experience success and self-confidence, enjoy the fun of teachers’ work, feel the charm of education, and enhance the sense of teaching efficacy, so as to enable teachers to actively optimize their professional values, complete education and teaching work efficiently, and promote the improvement of work performance. In addition, we should also create a safe and stable environment, create a good interpersonal atmosphere, help teachers form positive professional values and improve teachers’ job satisfaction.

Third, according to the findings of this study, the utilization of support and subjective support were significantly associated with the job satisfaction of university teachers. The university management department should promote humanized management. In addition to providing direct objective material support to university teachers, they should also actively communicate with university teachers and promote the communication between leaders and teachers, teachers and teachers. Establish and enhance the emotional friendship between each other, so that university teachers can feel multi-level support, make full use of these supports, and constantly make social support enter their inner world, so as to improve job satisfaction.

There are also some limitations in this study that should be borne in mind when assessing the value of the findings. First, the use of cross-sectional research design makes it difficult to infer causal relationships among the variables; thus, future researchers could employ longitudinal or experimental study design to test our hypotheses. Second, we mainly relied on self-reports, which may lead to reliance on self-awareness and reported biases (72). Future researchers could test the effect with more varied procedures. Third, although multiple factors including family, work values, and social support were integrated into models, we focused only on the unique effect of each factor and did not explore interaction effects. Future researchers could investigate these interaction effects among these factors. Finally, this study only identified gender differences, but failed to further explore the mechanisms underlying these differences through subgroup analyses (e.g., stratified by academic rank or years of service). Cultural factors, work-family balance pressure, or differences in rank distribution may contribute to these results. Future research could further explore the mechanisms behind gender differences and provide more comprehensive insight into practical improvements.

## Data Availability

The original contributions presented in the study are included in the article/supplementary material. Further inquiries can be directed to the corresponding author.
